# Robust soybean seed yield estimation using high-throughput ground robot videos

**DOI:** 10.3389/fpls.2025.1554193

**Published:** 2025-03-31

**Authors:** Jiale Feng, Samuel W. Blair, Timilehin T. Ayanlade, Aditya Balu, Baskar Ganapathysubramanian, Arti Singh, Soumik Sarkar, Asheesh K. Singh

**Affiliations:** ^1^ Department of Computer Science, Iowa State University, Ames, IA, United States; ^2^ Department of Agronomy, Iowa State University, Ames, IA, United States; ^3^ Department of Mechanical Engineering, Iowa State University, Ames, IA, United States

**Keywords:** yield estimation, soybean seed counting, plant phenotyping, deep learning, computer vision

## Abstract

We present a novel method for soybean [*Glycine max* (L.) Merr.] yield estimation leveraging high-throughput seed counting via computer vision and deep learning techniques. Traditional methods for collecting yield data are labor-intensive, costly, and prone to equipment failures at critical data collection times and require transportation of equipment across field sites. Computer vision, the field of teaching computers to interpret visual data, allows us to extract detailed yield information directly from images. By treating it as a computer vision task, we report a more efficient alternative, employing a ground robot equipped with fisheye cameras to capture comprehensive videos of soybean plots from which images are extracted in a variety of development programs. These images are processed through the P2PNet-Yield model, a deep learning framework, where we combined a feature extraction module (the backbone of the P2PNet-Soy) and a yield regression module to estimate seed yields of soybean plots. Our results are built on 2 years of yield testing plot data—8,500 plots in 2021 and 650 plots in 2023. With these datasets, our approach incorporates several innovations to further improve the accuracy and generalizability of the seed counting and yield estimation architecture, such as the fisheye image correction and data augmentation with random sensor effects. The P2PNet-Yield model achieved a genotype ranking accuracy score of up to 83%. It demonstrates up to a 32% reduction in time to collect yield data as well as costs associated with traditional yield estimation, offering a scalable solution for breeding programs and agricultural productivity enhancement.

## 1 Introduction

Soybean [*Glycine max* (L.) Merr.] is one of the most important crops in the world. It is a legume that serves as an excellent source of high protein and oil for both humans and livestock (Medic et al., 2014). For soybean cultivar development by breeders, seed yield is one of the most critical traits for making selections and cultivar release decisions. Current methods for gathering yield data on experimental lines and candidate varieties require expensive machinery, extensive travel, and prolonged equipment operation. These are all prone to equipment breakdowns and incur high maintenance costs (Singh et al., 2021a). The data collection procedure involves harvesting thousands, and potentially hundreds of thousands, of plots across multiple locations. These economic and time burdens have motivated researchers to explore new techniques, such as remote sensing and ground robot systems, as well as machine learning (ML) and computer vision (CV) methods, to estimate yield in a more efficient and cost-effective manner.

Significant improvements in machine learning and computer vision have given breeders new approaches for cultivar development using remote sensing platforms and ground robot systems (Singh et al., 2021b; Ma et al., 2023; Chen et al., 2023; Sarkar et al., 2024). On the one hand, remote sensing platforms such as uncrewed aerial systems (UAS) offer data collection and phenotyping tools that can be used to estimate yield (Herr et al., 2023). On the other hand, ground robot systems utilize ground data like field images or LiDAR data for yield prediction. This paper focuses on using ground data to estimate yield. For the ground robot systems, the detection and quantification of plant organs can serve as a proxy for crop yield. Recent developments in plant organ detection have been applied successfully in various crops, such as apple orchards for fruit detection (Bargoti and Underwood, 2017; Kang and Chen, 2020), grape vineyards for grape and shoot detection (Grimm et al., 2019; Guadagna et al., 2023), sweet pepper fruits (Sa et al., 2016), and peanuts (Puhl et al., 2021).

Applying similar detection techniques to soybean pods offers noteworthy value to soybean breeders for soybean yield estimation. Soybean pod count has been shown to strongly correlate with yield (McGuire et al., 2021). However, unlike other crops, soybean pods present unique challenges due to their smaller size and the occlusion caused by dense foliage. As sensor technology and ML tools advance, methods for estimating soybean pod counts as yield are continually emerging. There have been several studies using deep learning models and computer vision approaches to detect and quantify soybean pods. Some used RGB imagery with a black or white background to image pods from mature soybean plants (Xiang et al., 2023; Zhang et al., 2023; Yu et al., 2024), while others used similar background techniques but with potted plants (Lu et al., 2022; He et al., 2023). Notably, Riera et al. (2021) proposed a deep multiview image fusion architecture that minimizes human intervention and enhances yield estimation accuracy. This method uses a deep learning (DL) framework to detect soybean pods and estimate yield from RGB images collected by a mobile ground phenotyping unit. It improves the efficiency of yield testing trials and facilitates timely data collection for breeding decisions. Additionally, this approach can be integrated with drone-based phenotyping to further reduce labor and time in breeding programs (Li et al., 2024). Besides this, soybean researchers used three-dimensional imaging technologies such as LiDAR to create new phenotyping tools for soybean breeding purposes (Young et al., 2023, 2024). A similar technique was used for soybean pod detection by employing a depth camera to render a three-dimensional view of an entire soybean plant (Mathew et al., 2023). This method uses imagery to detect the distance from the plant to the camera, creating a three-dimensional heat map that is then used to estimate pod count. The methods mentioned above are all based on soybean pod detection or pod counting and use that as a proxy for yield. They have demonstrated significant potential for accurately estimating pod count and, in some cases, providing yield ranking estimates.

Nevertheless, a better seed yield estimate will be seed count on standing plants. A stronger correlation between soybean yield and the number of seeds (*r* = 0.92) was reported (Wei and Molin, 2020). Researchers have developed some soybean seed detection models (Uzal et al., 2018; Li et al., 2019). A significant limitation of these works is their reliance on imagery of pods with either black or white backgrounds. They have yet to be implemented in a high-throughput data collection manner in a breeding plot field environment. In these works, they consider seed detection as a common object detection problem that involves locating and identifying each individual separately. Unlike object detection, however, a more specific task called crowd counting aims to directly locate target objects and estimate their count in one shot. Crowd counting approaches often perform much better in densely populated scenes than regular object detection methods. Considering that a soybean field is a crowded scene, researchers from the University of Tokyo treated the seed yield estimation as a crowd-counting problem (Zhao et al., 2023). They proposed the P2PNet-Soy model, which was extended from P2PNet (Song et al., 2021), for soybean seed counting. Key improvements in this model include the integration of k-d tree postprocessing, multiscale multireception field feature extraction, and attention mechanisms, significantly reducing the mean absolute error (MAE). The study underscores the importance of considering high-level and low-level features to enhance model accuracy, demonstrating substantial improvements in seed counting and localization performance. However, it is yet needed to address the scale of phenotyping and decision-making in a breeding program that requires high accuracy.

Overall, the main gaps to address in the development of a seamless phenotyping method for seed yield estimation in a breeding program are a) a large number of plots across different tests, b) varying genetic variation among plant materials, c) a field environment that is impacted by multiple weather elements complicating data collection, and d) DL models with improved accuracy and generalizability. Challenges exist in soybean seed detection and quantification with computer vision and machine learning methods. Errors in computer vision tasks can be compounded by background noise, object occlusion, cluttered image environments, variable lighting, and weather conditions, among other factors. In this paper, we propose a method for non-destructively estimating soybean seed yield using computer vision and deep learning. We first capture video data from a ground robot, which will be segmented into individual video frames, i.e., images. We then train a DL model that detects and quantifies seeds captured in these images and uses the estimated seed count to rank plots for breeding decisions. Yield ranking, as opposed to direct computation, provides breeders with an efficient way to compare different experimental lines and candidate varieties, including their relative performance to established checks. Our proposed approach is a great alternative solution to combine yield. It is especially practical and efficient when ground-robot-based imaging with our yield estimation model (P2PNet-Yield) can be used to save time and resources, or when absolute yield measurements are not feasible due to time constraints or machine breakdowns.

Here are some highlights of our work. First of all, we conducted a 3-year field experiment for data collection, which covers varying genetic variation and field environment. During data collection, we ensured that every side of every detected plant was imaged so seeds occluded by plants or other pods from one side were still captured on the opposite side. Secondly, as for the design of our deep learning architecture, we proposed a yield estimation framework called P2PNet-Yield, which is based on P2PNet-Soy (Zhao et al., 2023) and the two-stage architecture of Riera et al. (2021). Thirdly, several strategies were adopted in our model training. For example, to enhance the generalizability of the seed detection model (P2PNet-Soy), we trained it using image data captured under various imaging conditions with different camera sensor effects applied. Last but not least, our method combines high-throughput phenotyping using ground-robot-based imaging for seed detection and quantification. It can efficiently estimate and rank soybean yield for plant breeding decisions to select varieties. The genotype ranking based on seed counting and yield estimation results was conducted to evaluate the performance of our work.

To summarize, the main contributions of our work are a) the creation of a large-scale image dataset of soybean plants with various genotypic traits collected over 3 years (2021 to 2023, though the 2022 data were not used in this work); b) the development of strategies to improve the accuracy of the seed-counting model trained on fisheye data, including fisheye image correction, data augmentation with random sensor effects, and a spatial adjustment method to account for environmental variations; and c) the creation of a yield estimation architecture (P2PNet-Yield) that combines the backbone of the seed counting model (P2PNet-Soy) with a custom yield estimation regressor.

## 2 Materials and methods

### 2.1 Field experiments and data collection

#### 2.1.1 Field experiments

Field experiments consisted of plant breeding trials in the ISU soybean breeding program. We collected data from F_5_ and F_7_ filial generation yield plots (Singh et al., 2021a). Phenotyping was done in the advanced soybean yield trials near Boone, IA (42.020–93.773, 339 meters above sea level). Robot video data were captured from two fields: a) in 2021—filial generation 5 (F_5_), number of yield trials = 8,500; and b) in 2023—filial generation 7 (F_7_), number of yield trials = 650. Data from the 2021 F_5_ field were used for the training and testing of the seed detection model, while the 2023 F_7_ data were used for yield prediction and ranking. In each year, yield trials were in a two-row configuration with a row spacing of 0.76 m, a seed-to-seed spacing of 3.68 cm, and 0.91-m alleyways. Plot lengths were 2.13 m in F_5_ and 5.18 m in the F_7_ generation. The plots in these experiments represent a genetically diverse collection of breeding populations representing elite and plant introduction parental stocks (Singh et al., 2021c).

Each year, soybean plots were seeded on a field that had bulk maize (*Zea mays* L.) the previous year. Standard ground preparation methods were practiced. Each field was treated with a post-planting herbicide a month after planting. In addition to chemical weed control, manual weed control was done by routinely visiting the plots to remove weeds.

#### 2.1.2 Data spatial adjustment

**Figure 1:**
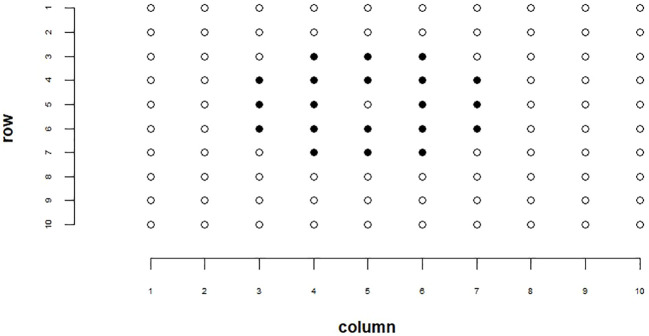
Example of our spatial adjustment grid pattern. Highlighted cells represent plots used in the spatial adjustment. The center cell that is not highlighted represents the cell being adjusted.


Spatial adjustment techniques can be applied to breeding plots to help account for environmental variations such as soil properties and reduce non-genetic variability in our data (Carroll et al., 2024). To account for environmental variation in our analysis, we employed the moving grid adjustment method provided by the mvngGrAd package (Technow, 2015). This adjustment was performed on our ground truth plot yields, estimated total seed counts, and estimated yields for each plot. Genotype ranking results reported in this paper were conducted on spatially adjusted data. Methods for estimated total seed count and estimated yields are explained in later sections. The spatial adjustment method involves adjusting a plot’s value based on the values of its neighboring plots within a defined grid. This grid can be adjusted relative to the plot of interest in the 0°, 90°, 180°, and 270° directions based on user definition and need. For our spatial adjustment pattern, we utilized a 5 × 5 grid, excluding the corner plots and the center plot. This grid configuration is depicted in **Figure 1**. The movingGrid() function, part of the mvngGrAd package, performs the spatial adjustment by calculating moving means for each plot based on this grid of neighboring plots. The adjusted phenotypic value *p_i,_*_adj_ is calculated using Equation 1, which is shown below:

(1) $$p_{i,\text{adj}}=p_{i,\text{obs}}-b\left(x_{i}-\bar{x}\right)$$

where:

-
$p_{i,\text{adj}}$ is the adjusted phenotypic value for entry *i*.
-
$p_{i,\text{obs}}$ is the observed phenotypic value for entry *i*.
- *b* is the coefficient representing the relationship between the growing conditions and the observed phenotypic value.
- *x_i_* is the moving mean phenotypic value for entry *i*, calculated as the mean of the cells within the grid around entry *i*.
-
$\bar{x}$ is the overall mean of the moving means *x_i_*.

#### 2.1.3 Ground-robot-based image data collection

**Figure 2:**
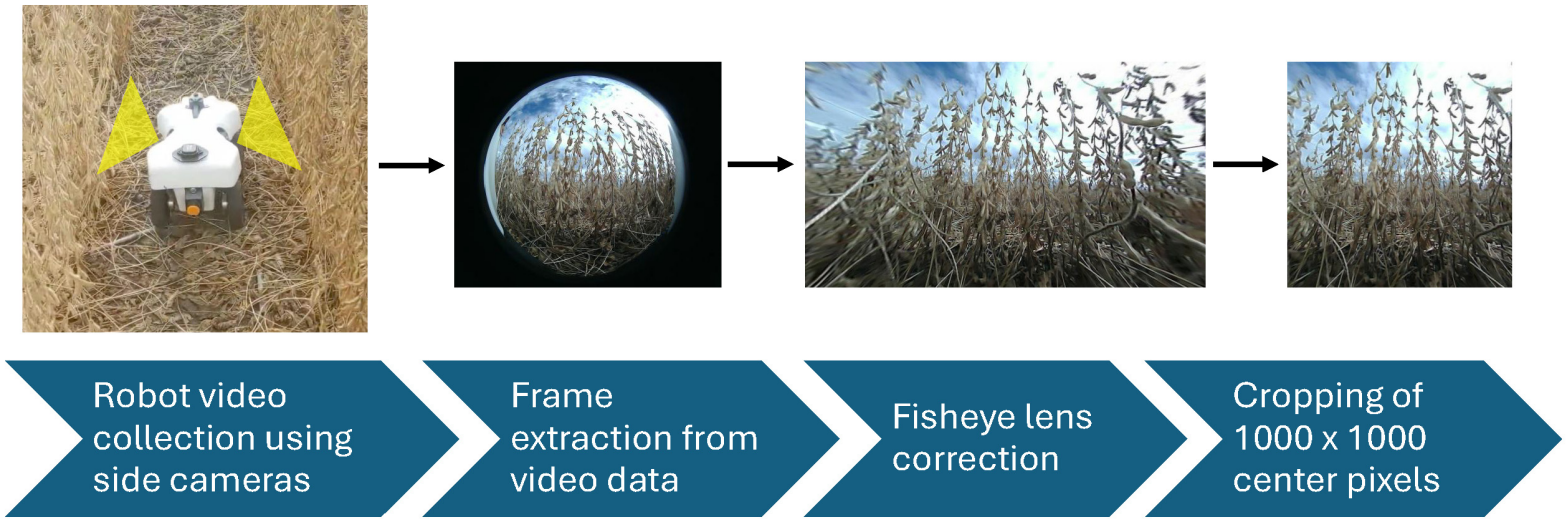
This figure demonstrates the pipeline from in-field data collection and the postprocessing needed for use in our P2PNet-Yield model. The first figure shows our TerraSentia robot operating in a mature soybean field. As the robot moves through the field, the two side-mounted cameras collect fisheye video data. Individual frames are then extracted, corrected for fisheye distortion, and cropped to remove blurry edges.

**Figure 3:**
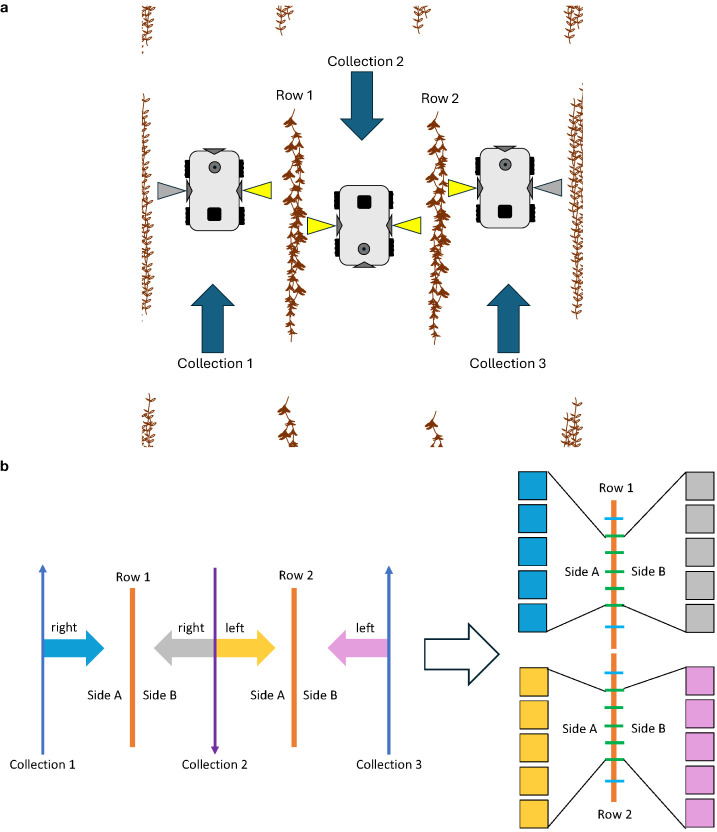
**(a)** The data collection process for a single plot. The yellow-highlighted cameras represent video sections belonging to the center plot. Gray-highlighted cameras represent video of the other plots. Three collections in total are needed to fully capture a single plot. Automatic postprocessing with Python scripts organized these videos into their respective plots. Arrows represent the direction of robot movement. **(b)** The image data sampling process for a single plot. Each row was equidistantly divided into eight sections using seven splitters, with images from the middle five splitters chosen for analysis. (The first and last splitters were excluded.) The two rows of the same plot were connected and treated as one single row, resulting in 10 images per side and 20 images per plot.

The robot used in this experiment was the TerraSentia, developed by EarthSense (EarthSense, Champaign, USA) (McGuire et al., 2021). This robotic platform is equipped with two side-facing cameras, a forward-facing camera, an upward-facing camera, vertical and horizontal LiDAR sensors, and an RTK GPS. **Figure 2** shows the robot operating in a field. The video cameras on this robot were fitted with fisheye lenses, ensuring comprehensive capture of the soybean plants from the base to the top. For this experiment, the two side-facing cameras were utilized. Data were collected as side-view videos of soybean plots after the entire field had reached full physiological maturity (stage R8) (Fehr et al., 1971). Video data were collected by manually navigating the robot between each row of soybeans (**Figure 3a**). Videos were recorded at a resolution of 1,920 × 1,080 pixels. Each traverse of the robot through the field resulted in a continuous video recording, which we will refer to henceforth as a “collection.” Each collection began at the start of a pass and ended once the robot reached the end of that pass. The robot was maneuvered in a serpentine pattern to ensure imaging of both sides of every row in each plot. This approach aimed to capture pods that might have been obstructed from one side but were visible from the other. The dual side-mounted cameras on the TerraSentia allowed us to achieve this with a minimum number of collections. Although a single collection captures multiple plots, three collections were needed to fully capture any one plot.

#### 2.1.4 Data preprocessing

Following the collection of video data, individual frames were extracted from each video using Python scripts. As previously mentioned, these frames were captured using fisheye lenses, which introduced distortion artifacts. To achieve accurate seed counts and yield estimations, it was essential to remove these distortions. An OpenCV (Bradski, 2020) tool was employed to calibrate the images, correcting for fisheye lens distortion. Calibration was conducted using a standard checkerboard pattern, with multiple images captured from varying angles and distances to ensure precise calibration. This was done in accordance with a pre-existing method (Jiang, 2017). Through this calibration process, the intrinsic camera parameters of the fisheye camera were obtained: the focal length (*f_x_*, *f_y_*) in pixels is (410,410), and the principal point (*p_x_, p_y_*) in pixels is (383,526). Using these parameters, the fisheye distortion was effectively corrected (see **Figure 2**). After correcting the fisheye distortion, the edges along the sides, as well as the top and bottom of the images, were left blurry and sometimes still distorted. A central area measuring 1,000 × 1,000 pixels was then cropped. It was later utilized for image annotation, training, and total seed count (TSC) estimations. This ensured that the outside blurred regions were removed, mitigating the background noise (see **Figure 2**).

Each calibrated frame was then assigned to its respective plot using the on-board vertical LiDAR sensor to detect the start and stop points of each plot. These start and stop points were generated by EarthSense’s proprietary data processing tools. They were provided as a CSV file containing a series of time points representing the start and stop times for each plot. Each frame in the video was associated with a corresponding time point. Python scripts were used to extract these time points and their associated frames, organizing each set of images into their respective plots. We manually proofed the accuracy of the plot segmentation data provided by EarthSense by randomly checking approximately 50% of the images, and no discrepancies were found.

Once organized by plot, the images were further sorted by their respective row and side assignments. Specifically, the plot images were organized first by plot name (range, pass), then by row number (1 or 2), and by row side (A or B). This resulted in four sets of images, each set containing approximately 100 images, or approximately 400 images per plot. In total, 338,793 images were generated from the F_7_ generation material in 2023. Plots from 2023 were used to test the accuracy of the seed count and yield estimation model in ranking genotype yield.

Given that the number of images per plot could be large, a selection process was employed for seed counting and yield estimation. As illustrated in **Figure 3b**, each row was equidistantly divided into eight sections using seven splitters, with images from the middle five splitters chosen for analysis. The two rows of the same plot were treated as a single row, resulting in 10 images per side and 20 images per plot. These 20 sample images were evenly distributed across the plot, containing the most representative information. The first and last splitters were excluded from the analysis, as they were too close to the start or end of each row and occasionally did not contain any plants.

#### 2.1.5 Seed count annotations and ground truth yield data collection

**Figure 4:**
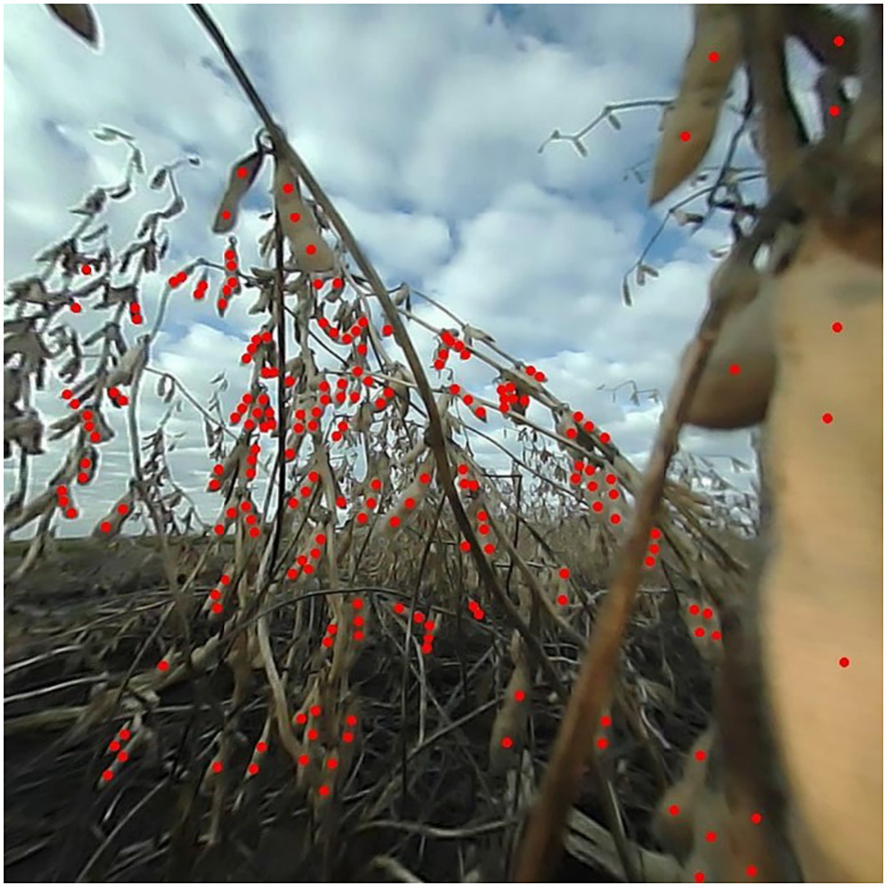
Example of an expertly annotated image. All seeds clearly discernible to the naked eye were annotated using point annotations. This image illustrates a calibrated and cropped frame with soybean seeds annotated in red.

From the set of images captured from the F_5_ generation material in 2021, a subset of 1,200 images was randomly selected for seed annotation. Expert raters conducted these annotations, marking only visible soybean seeds in each image using point annotations. The annotations were facilitated by the Label Studio software (HumanSignal, Inc, 2023). Only seeds that were clearly discernible by human raters were annotated. Seeds that were too indistinct to separate and the ones located in background plots were excluded (see **Figure 4**). This subset of images was taken at any time of day from 8:00 a.m. to 6:00 p.m. It represents a range of weather and lighting conditions, as well as varying levels of soybean lodging and occlusion. The genotypes captured in these images exhibit diverse pubescence and pod wall colors. The images were selected across the plot, ensuring the inclusion of both seed-rich and seed-scarce images. This strategy enhances the model’s ability to detect seeds under a wide variety of conditions, thereby improving its generalizability.

A set of ground truth data was collected for yield. Ground truth yield data for each plot were obtained using either a Zurn (Zürn Harvesting, Schöntal-Westernhausen, Baden-Württemberg, Germany) or Almaco (Almaco, Nevada, IA, USA) plot combine, which provided seed yield measurements in kilograms for each plot. Ground truth yield data were collected for all plots imaged by the TerraSentia (see Section 2.1.3). To ensure consistency in yield measurements across fields with varying plot sizes, yields were adjusted to 13% moisture and converted to metric tons/hectare (MT/ha) for each harvested plot.

### 2.2 Seed counting on fisheye data

The undistortion process applied to the fisheye images resulted in more consistent patterns of soybean seeds. This consistency made it easier to train the feature extraction backbone of the seed counting model, P2PNet-Soy (Zhao et al., 2023). The model’s pretrained weights were utilized to further enhance training efficiency. We trained the P2PNet-Soy model on our corrected image data to help it learn feature maps that contain relevant information about the seeds.

To prepare the training datasets, we augmented our original corrected image dataset by applying random camera sensor effects, such as noise, blurring, chromatic aberration, and exposure adjustment (Carlson et al., 2018). The purpose of this data augmentation strategy was to reduce the differences between images captured by different cameras in varying environments. For instance, noise was added to simulate the common artifacts found in low-quality images due to sensor limitations. Blurring could reduce edge sharpness to account for motion or focus issues. Chromatic aberration was introduced to replicate the color fringing caused by lens imperfections, and exposure adjustments helped simulate changes in lighting conditions or camera quality.

The data augmentation process started with our original dataset. With data augmentation, we obtained the augmented datasets, which include both the original images and those modified by these camera sensor effects. Each original image has one augmented version with various sensor effects applied. Instead of uniformly applying these effects to all images, we introduced variability by randomly adjusting the intensities of each effect. These augmentations were implemented using a tool developed by Carlson et al. (2018), which can automatically select random parameter settings for each effect. By incorporating these augmentations, we aimed to make our model more robust to variations in image quality across different cameras and environments. The improvements brought about by this augmentation technique are discussed in Section 3.1.

With the augmented data, we prepared three datasets for training: UTokyo, ISU2021, and ISU2021-Aug. The UTokyo dataset includes 126 images for training and 27 for evaluation, which was provided by the University of Tokyo team who introduced P2PNet-Soy (Zhao et al., 2023). The ISU2021 dataset comprises 1,200 annotated images from the 2021 F_5_ field, divided into two subsets: 1,007 images for training and 193 for evaluation. The ISU2021-Aug dataset was created by applying camera sensor effects to our ISU2021 dataset and consisted of augmented versions of the 1,007 training images from ISU2021.

We trained the seed counting model on four different combinations of these three datasets to identify the best model for seed detection and counting. These four combinations were ISU_NO_AUG (using only the ISU2021 data), MIX_NO_AUG (using ISU2021 and UTokyo), ISU_AUG (using ISU2021 and ISU2021-Aug), and MIX_AUG (using ISU2021, ISU2021-Aug, and UTokyo). During training, the weights from the original P2PNet-Soy model were employed, and most hyperparameters were kept at their default settings. For each combination, the model was trained for 100 epochs. The model was validated using the mean squared error (MSE), mean absolute error (MAE), and mean absolute percentage error (MAPE), which can be found in Section 3.1.

### 2.3 Yield estimation architecture

**Figure 5:**
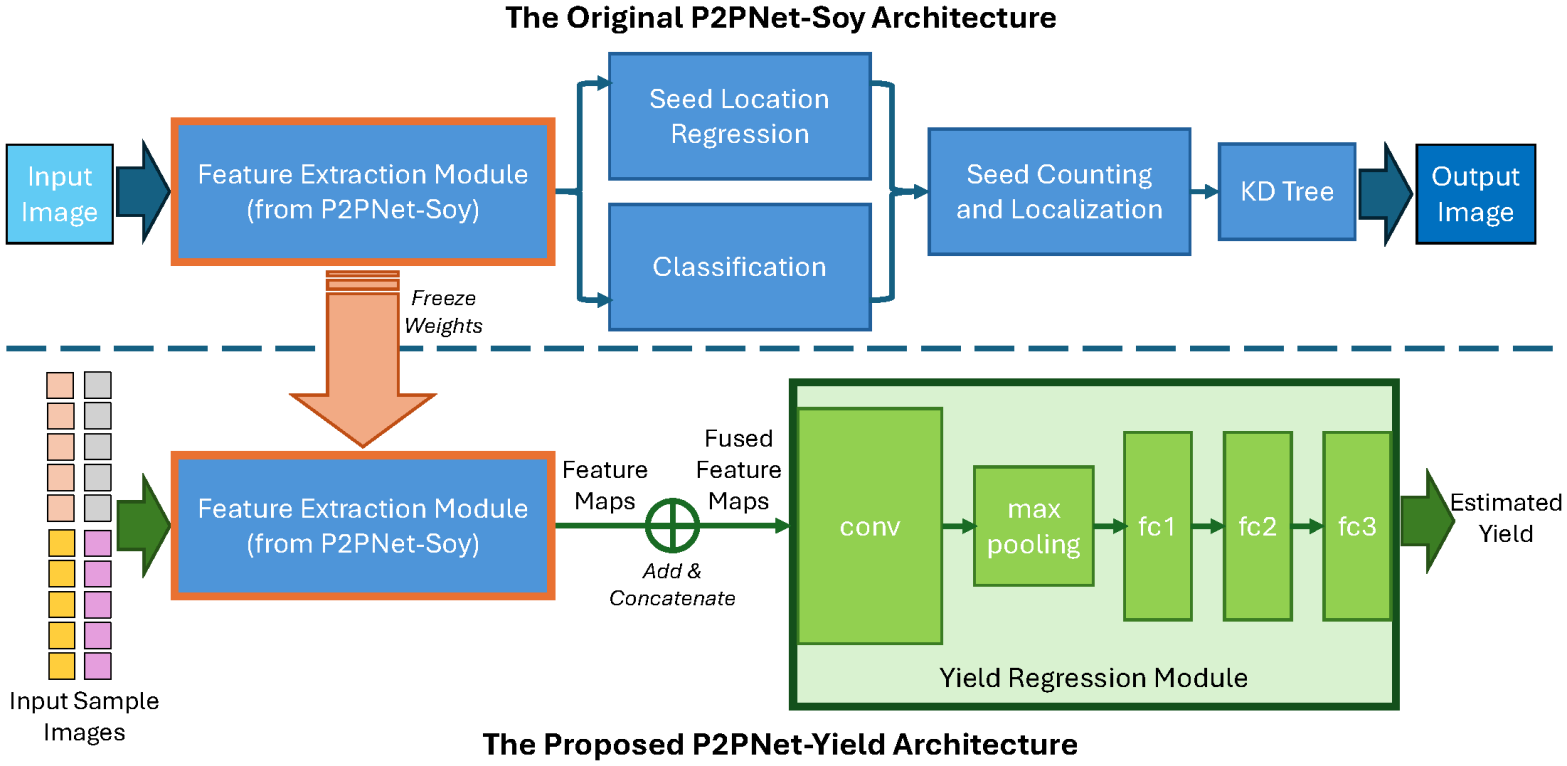
Our architecture, P2PNet-Yield, for soybean yield estimation. Training consists of two phases: first, training the P2PNet-Soy model so that its backbone (used as our feature extraction module) can extract useful information related to soybean seeds in the foreground; second, training our yield regression module to estimate yield values from the output feature maps of the feature extraction module.

Inspired by previous works (Riera et al., 2021), our DL architecture for yield estimation, named P2PNetYield, consists of two modules: the feature extraction module and the yield regression module. The architecture is depicted in **Figure 5**. The backbone of the seed counting model (P2PNet-Soy) serves as the feature extraction module. The feature map extracted by this module contains valuable information that can be used for seed detection. For each plot, we applied our feature extraction module to the 20 sample images selected (as discussed in Section 2.1.4), obtaining 20 feature maps.

Once these feature maps were obtained, the next task was to predict yield using these feature maps. To fuse information from these feature maps, we summed the 10 feature maps from the same side and then concatenated them. The fused feature map was then fed into the yield regression module. The yield regression module consists of one convolution layer (conv) followed by a max-pooling layer, which is then flattened and followed by three fully connected layers (fc1, fc2, and fc3). The output of the yield regression module is the yield (in MT/ha) for the plot. During training, the layers in the feature extraction module were frozen. The batch size was set to 8, and the models were trained for 50 epochs. We adopted the Adam optimizer (Kingma and Ba, 2014) and used element-wise mean squared error as our loss function.

As described above, seed counts do not serve as a direct intermediate parameter in our yield estimation architecture. Our approach begins by fine-tuning P2PNet-Soy to accurately detect and count soybean seeds, ensuring that its backbone effectively extracts meaningful features (as discussed in Section 2.2). We then freeze the backbone’s weights and introduce our yield regression module, forming the P2PNet-Yield architecture. This regression module directly utilizes the extracted feature map, rather than seed counts, to predict yield. Therefore, while seed counts are the output of P2PNet-Soy, they are neither our final nor intermediate output of P2PNet-Yield.

The primary goal of this work is to provide an accurate yield ranking for guiding crop breeding programs, rather than chasing very precise seed count or yield values. Therefore, using only 20 images per plot may be insufficient for precisely estimating the total seed count and yield but should be enough for yield ranking prediction. The final yield ranking performance of P2PNet-Yield depends more on the feature extraction capability than on absolute seed counts. In other words, it is about how well the model (i.e., the backbone of P2PNet-Soy) can extract meaningful features that correlate with yield ranking (such as the distribution and size of the seeds, rather than only the absolute counts).

## 3 Results

The training and testing experiments of our deep learning architecture utilized the PyTorch library version 1.8.0 with CUDA 11.1 support for an NVIDIA GPU. For all the results presented in this section, we used an NVIDIA A100 GPU with 80 GB VRAM running on a CPU with Intel Skylake Xeon processors with 512 GB RAM.

To evaluate our approach and explore possible improvement, we conducted a multistage experiment: 1) fine-tuning P2PNet-Soy (Section 3.1), 2) evaluating yield ranking accuracy (Section 3.2), and 3) investigating performance improvement (Section 3.3).

### 3.1 Seed counting

We first assessed the performance of the seed counting model (P2PNet-Soy) on our data in seed detection and counting, ensuring its backbone was suitable for our yield prediction architecture. In this stage, annotated seed counts in 1,200 images randomly selected from the 2021 F_5_ data were considered ground truth (Section 2.1.5). Our test set consists of 100 mixed images, with a 5:1 ratio of ISU2021 to UTokyo images, ensuring diverse evaluation conditions. All test images were manually annotated and counted. No augmented images were included in this test dataset. The goal of the evaluation in this stage was to see if the model’s ability to generalize real-world data is improved.

**Figure 6:**
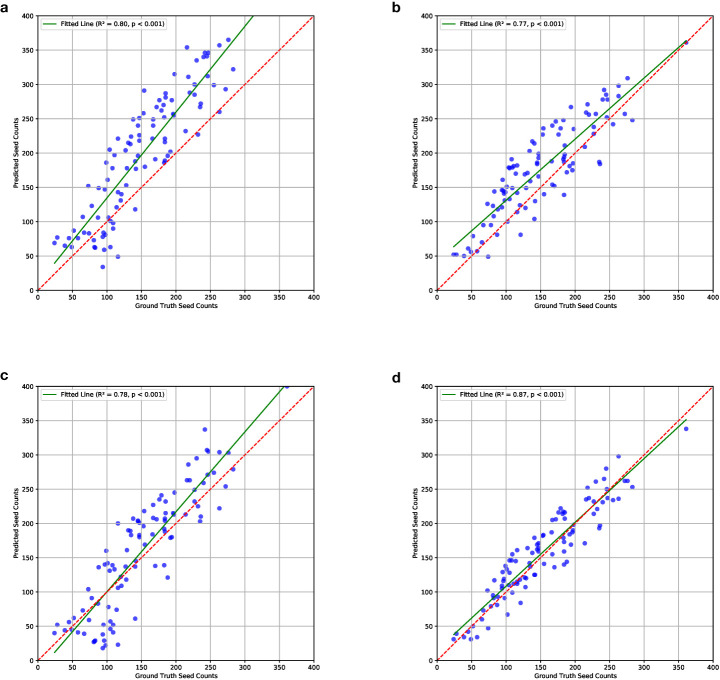
Correlations between ground truth and estimated seed counts of models trained on different combinations of the datasets. The combination details can be found in Section 2.2. Results show that the model trained on mixed datasets with data augmentation performs the best. **(a)** ISU_NO_AUG; **(b)** MIX_NO_AUG; **(c)** ISU_AUG; **(d)** MIX_AUG.

In our experiments, we noticed that the P2PNet-Soy model trained on our corrected fisheye images without any data augmentation did not generalize well and showed overcounting issues during testing (as shown in **Figure 6A**). Specifically, the P2PNet-Soy model struggled to distinguish between foreground and background soybean plants, leading to persistent seed detection in the background. This was likely due to differences in cameras and imaging conditions across the test datasets, which included images randomly selected from both our datasets and the one from the P2PNet-Soy team.

With data augmentation utilizing camera sensor effects, the performance of the seed counting model was improved. As discussed in Section 2.2, MSE, MAE, and MAPE were calculated to evaluate the fine-tuned P2PNet-Soy model trained on different combinations of the training datasets. For all three metrics, augmentation improved the model performance, i.e., ISU_AUG was better than ISU_NO_AUG, and MIX_AUG was better than MIX_NO_AUG (**Table 1**). Larger datasets with more environmental variations performed better than smaller data, i.e., MIX_NO_AUG was better than ISU_NO_AUG, and MIX_AUG was better than ISU_AUG (**Table 1**). The MSE, MAE, and MAPE were lowest for MIX_AUG. Meanwhile, MIX_NO_AUG and ISU_AUG had similar performance with slightly better MSE and MAE for ISU_NO_AUG (**Table 1**).

**Table 1:** Testing results (MSE, MAE and MAPE) of the models trained on different combinations of the datasets.

Combination	MSE	MAE	MAPE (%)
ISU_NO_AUG	4,277.83	54.93	41.26
MIX_NO_AUG	1,734.11	34.89	28.72
ISU_AUG	1,858.16	36.26	28.64
MIX_AUG	596.94	20.54	15.50

These results were visualized through correlation plots that plot the ground truth and estimated counts for each of the four dataset combinations (**Figure 6**). The correlations from ISU_NO_AUG showed an upward bias in the estimated counts, particularly at higher seed count values (*R* ^2^ value of 0.80, **Figure 6a**). The ISU_AUG had less bias, although still trending of overestimating at higher seed count values, but did not show a tighter fit to the regression line (*R* ^2^ value of 0.87, **Figure 6c**). The MIX_NO_AUG tended to overestimate at lower values (*R* ^2^ value of 0.77, **Figure 6b**). The best fit was noted for the MIX_AUG dataset combination with the highest *R* ^2^ (*R* ^2^ value of 0.87, **Figure 6d**). The *p*-values of these four combinations were all smaller than 0.001. This confirms that there is a significant linear relationship between the ground truth and predicted seed counts. The plots of residuals with ground truth seed counts, which can be found in the **Supplementary Materials**, showed that the model trained on MIX_AUG, i.e., mixed datasets with data augmentation, performed the best.

The results indicated that the model trained on mixed datasets with data augmentation performed best as per MSE, MAE, and MAPE; correlations; and residual plots. Data augmentation using camera sensor effects effectively reduced overcounting. Errors were further minimized by combining data from different sources.

### 3.2 Applications in plant breeding and selection

The application of P2PNet-Soy (Zhao et al., 2023) and our P2PNet-Yield model was tested in two scenarios to demonstrate their usefulness in a variety of development plant breeding programs. Our evaluation focused on yield ranking accuracy, not absolute yield estimation. Both models’ backbones used the weights trained on the MIX_AUG dataset combination.

In the first scenario, we used the P2PNet-Soy model for seed counting to assign ranks for experimental lines, followed by breeding selection decisions. The TSC of each plot here is not the actual seed count of the whole plot. We summed the seed counts detected by P2PNet-Soy across 20 images as a proxy for yield for each plot and evaluated yield ranking performance.

In the second scenario, we used the P2PNet-Yield model to estimate seed yield (MT/ha) and assigned ranks for the experimental lines to make breeding selection decisions. In other words, we directly fed the 20 sample images of each plot into our P2PNet-Yield model, which predicted a single estimated yield value for yield ranking.

**Figure 7:**
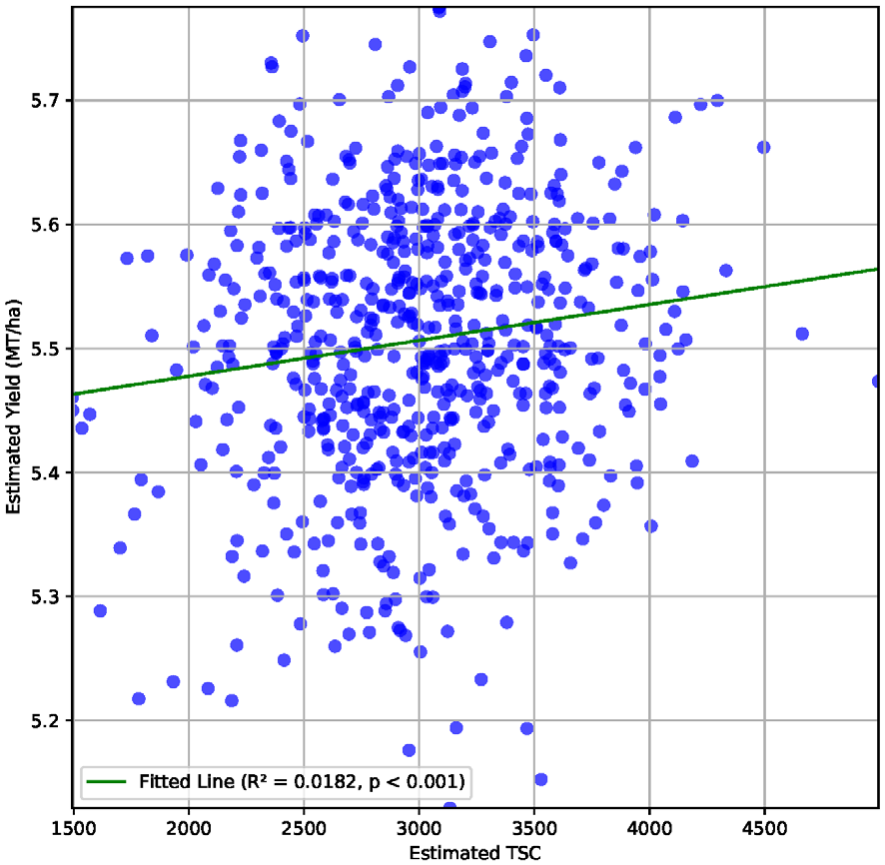
Correlation between estimated TSC and estimated yield (metric tons per hectare) for the 650 plots from the 2023 F_7_ field with a correlation coefficient of 0.14.

We report an *R* ^2^ value of 0.02 between estimated values of total seed counts from P2PNet-Soy and estimated yield from P2PNet-Yield (**Figure 7**). We evaluated three essential selection metrics—accuracy, sensitivity, and specificity—using selection thresholds of 30%, 20%, and 10%. To clarify how we define true positives (TP), false positives (FP), true negatives (TN), and false negatives (FN) in this context, here is an example. For a given selection threshold (e.g., 10%), the ground truth is the correct classification of top-performing vs. lower-performing genotypes/genetic materials: 1) TP: correctly identified top 10% genotypes; 2) FP: poor-performing genotypes incorrectly classified as top 10%; 3) TN: correctly identified lower 90% genotypes; an 4) FN: top-performing genotypes incorrectly classified as lower 90%. These metrics were calculated using spatially adjusted ground truth plot yields and spatially adjusted TSC from the 2023 F_7_ material, as these advanced yield tests were grown with two replications.

#### 3.2.1 Variety ranking and selections based on seed counting using P2PNet-Soy

**Figure 8:**
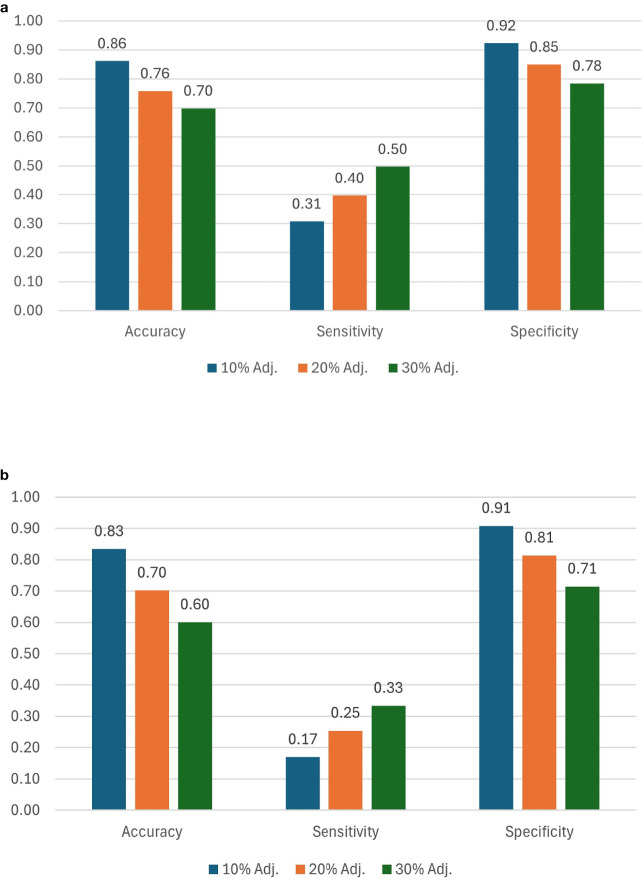
Ranking scores for 10%, 20%, and 30% selection thresholds using **(a)** TSC and **(b)** estimated yield. Scores are computed using spatially adjusted ground truth yields, TSC, and estimated yields.

**Figure 9:**
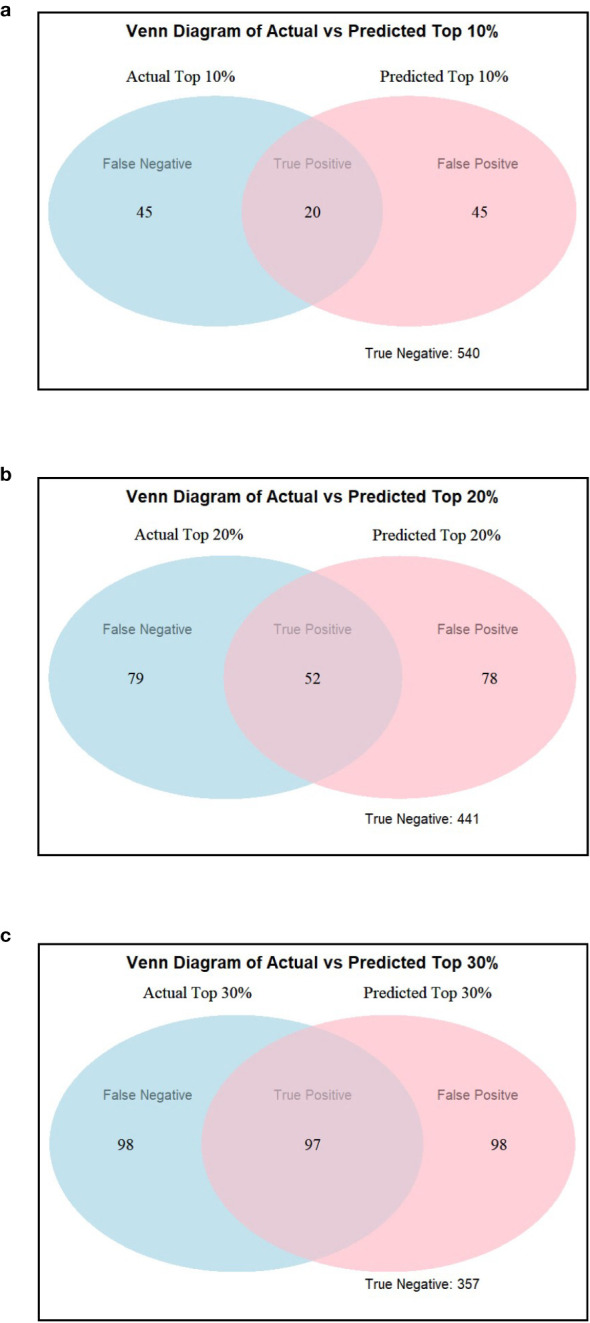
Venn diagrams showing actual versus estimated highest yielding lines using **(a)** 10%, **(b)** 20%, and **(c)** 30% selection threshold using TSC as an estimate for yield ranking.

Accuracy and specificity scores were relatively high across all three selection thresholds. We note that when the selection threshold becomes more stringent, accuracy and specificity scores increase, while the inverse is true for sensitivity. At 10%, 20%, and 30% selection cutoff, the accuracy values were 0.86, 0.76, and 0.70, respectively. Similarly, the specificity values were highest at a more stringent cutoff (0.92 at 10%, 0.85 at 20%, and 0.78 at 30%). In contrast, sensitivity scores progressively increased as the selection cutoffs were increased, i.e., 0.31 at 10%, 0.40 at 20%, and 0.50 at 30% (**Figure 8a**). The number of observations for TP and TN was the highest at the 10% cutoff, and the total number of correct classes (i.e., TP and TN) reduced at 20% and were the lowest at the 30% cutoff (**Table 2**; **Figure 9**). The FP and FN values were nearly identical within each selection threshold and increased from 10% to 30%.

**Table 2:** True-positive (TP), true-negative (TN), false-positive (FP), and false-negative (FN) values were calculated using a 10%, 20%, and 30% selection threshold based on estimated TSC and estimated yield ranking.

		10% Threshold	20% Threshold	30% Threshold
Estimated TSC	TP TN FP FN	20 540 45 45	52 441 78 79	97 357 98 98
Estimated yield	TP TN FP FN	11 531 54 54	33 423 97 97	65 325 130 130

#### 3.2.2 Variety ranking and selections based on yield estimation using P2PNet-Yield

In addition to the analysis based on estimated TSC, we also evaluated genotype ranking based on estimated yield. When using our proposed P2PNet-Yield model, we note that as the selection threshold becomes more stringent, accuracy and specificity scores increase while the inverse is true for sensitivity. Accuracy and specificity scores were high across all three selection thresholds. At 10%, 20%, and 30% selection cutoff, the accuracy values were 0.83, 0.70, and 0.60, respectively. Similarly, the specificity values were the highest at a more stringent cutoff (0.91 at 10%, 0.81 at 20%, and 0.71 at 30%). In contrast, sensitivity scores progressively increased as the selection cutoffs were increased, i.e., 0.17 at 10%, 0.25 at 20%, and 0.33 at 30% (**Figure 8b**). The number of observations for TP and TN was the highest at the 10% cutoff, and the total number of correct classes (i.e., TP and TN) reduced at 20% and were the lowest at the 30% cutoff (**Table 2**). The FP and FN values were nearly identical within each selection threshold and increased from 10% to 30%. Overall, the trends from the P2PNet-Yield results were similar to P2PNet-Soy.

### 3.3 P2PNet-Yield performance under optimal conditions

**Figure 10:**
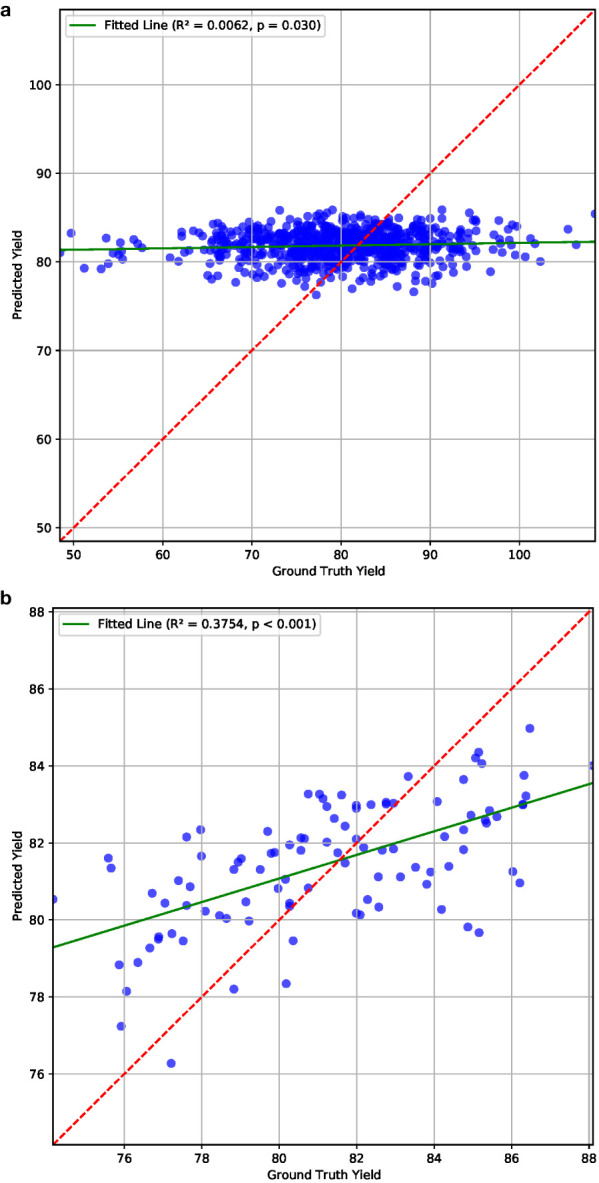
Yield estimation results on **(a)** all and **(b)** 100 selected plots from the 2023 dataset, which demonstrate the potential effectiveness of our P2PNet-Yield architecture in yield estimation under optimal conditions.

To assess the P2PNet-Yield model’s performance, we manually curated a subset of 200 plots from the 2023 F_7_ dataset which included 650 plots, i.e., we used 30% plots for this analysis. These plots did not have any anomalies, such as severe lodging and severe disease. During planting, harvest, and intermediate periods, notes were recorded for the plots exhibiting unique issues or characteristics, such as disease, lodging, or large gaps (*>*0.5 m gaps) of missing plants in the harvested rows. We also excluded plots with bad imaging caused by overexposure or camera misplacement. This resulted in a refined dataset comprising 100 plots for training and 100 plots for testing for our P2PNet-Yield model. The *R* ^2^ value was 0.38 between the estimated yield and ground truth yield for these plots and the MSE was 6.53. The *p*-value was smaller than 0.001, indicating that the relationship is highly statistically significant. No correlation was noted in the uncurated dataset (**Figure 10**). Although a manual selection was made to select high-quality plots from the dataset, these results demonstrated the potential efficacy of this architecture in yield estimation in high-quality field experiments.

## 4 Discussion

Much of the related research in yield or yield-related trait estimation relies on controlled imaging environments to estimate pod or seed counts (Li et al., 2019; Uzal et al., 2018; Zhang et al., 2023). These studies typically use imagery of harvested soybean pods against a uniform white or black background to train detection and quantification models. This approach simplifies seed detection by eliminating background noise and ensuring high-quality image. Other research expands on this by imaging entire soybean plants post-maturity (Xiang et al., 2023; Yu et al., 2024). In these cases, the entire plant is captured against a black background, allowing for pod detection across the whole plant.

Field-based studies often utilize small plots and relatively small datasets. Researchers have employed ground robots to extend this approach to larger plots (Riera et al., 2021; McGuire et al., 2021). These experiments focused on detecting soybean pods. Our research builds upon these efforts by introducing a novel pipeline for seed detection in a variety of development programs with field-based experiments, moving beyond controlled environment settings. While research has been done to present seed counting methods for yield estimation in a field environment, it was built on a small dataset (24 accessions; 374 images of individual plants) and lacked a high-throughput data collection method (Zhao et al., 2023). Our work is the first to implement seed counting as a high-throughput method for yield estimation in non-controlled environments and to analyze full-sized breeding plots for cultivar development purposes.

Utilizing small ground robots, our research presents a method of yield data collection that is approximately 50% faster than traditional two-row plot combine harvesting methods. These time savings can be multiplied by the number of robots deployed. Another benefit of operating small ground robots is the ease of operation versus traditional plot combines, which require much more intensive training to operate accurately and safely. The operation of these small robots is also much safer as injuries and fatalities caused by blind-spot accidents is a great concern in traditional plot combines (Ehlers and Field, 2017). These robots are also much cheaper to purchase and are easier and less costly to maintain and repair.

We proposed an innovative approach for estimating soybean seed yield using fisheye imagery data to detect seeds and estimate yield. Fisheye images, while providing comprehensive plant information, pose significant challenges due to distortion. To overcome this, we calibrated the fisheye camera to correct the images. Besides this, we improved the diversity of imaging conditions through data augmentation with camera sensor effects to enhance the generalization of the seed counting model. Our experiments with various dataset combinations revealed that models trained on mixed datasets with data augmentation yielded the best performance.

We designed an architecture integrating a feature extraction module and a yield regression module, demonstrating satisfactory yield estimation. Our results on seed counts and seed yield per area are similar to those previously achieved with pod counting using a ground robot (Riera et al., 2021). Our genotype ranking analysis indicates that both estimated TSC and estimated yield are effective for down-selecting poorly performing lines, which is similar to what was previously reported with image-based pod counting (Riera et al., 2021). Although both P2PNet-Soy and P2PNet-Yield methods exhibit low sensitivity scores—suggesting that both methods are suboptimal for identifying top-performing lines—high specificity scores confirm their utility in eliminating the poor-performing lines. This is particularly useful in early-stage yield trials. While TSC as a selection method performs marginally better in all three selection metrics than estimated yield (MT/ha) using the same dataset, our P2PNet-Yield model, when trained and tested on high-quality plots, exhibits a higher correlation between estimated and actual yields. We report the reasonable capability of our P2PNet-Yield model to estimate yield similar to the plot combine yield, at least in ranks, which is useful for a plant breeder to make selection decisions. These results also highlight the importance of high-quality data for optimal performance.

However, our work has several limitations and areas for improvement. First of all, the seed detection and counting model is highly dependent on image quality. The accuracy of seed counting deteriorates when parts of the soybean plants are obscured due to poor lighting or occlusion. This is because the feature extraction module may struggle to extract good feature maps with seed information in these challenging areas. Additionally, our use of fisheye lenses for comprehensive plant capture introduces challenges. The use of fisheye lenses, even after correction, leaves our images with distorted and blurry edges, requiring cropping to remove these regions. This process results in data loss from the very top and bottom of the images, limiting the model’s ability to detect seeds in these regions. Using a higher-resolution fisheye camera system would eliminate these blurry edges and would no longer require cropping and, therefore, no data loss. This could increase estimated TSC and yield (MT/ha) accuracy, improving yield ranking.

Another limitation arises during the seed annotation process, which was the inability to annotate every seed. Annotators were instructed to only make point annotations on seeds that were clearly discernible from other seeds. Due to an insufficient camera resolution, there were cases in which pods were clearly present in the plot of interest but seeds within those plots were not discernible. In other words, a pod may be visible in the image but the shapes and shadows that would indicate one seed from another may not have been detectable. In these cases, the seeds were not annotated. This likely is leading to a certain amount of seeds not being detected by our model and lowering the yield ranking accuracies. With a better camera system, these cases of undetectable seeds can be eliminated or at least minimized.

The data augmentation techniques through integrating random sensor effects are designed to enhance model robustness against common distortions observed in our dataset; however, they may not capture the full complexity of real-world variations. Our approach primarily focuses on replicating the key sensor artifacts present in our data, such as noise, blur, and exposure variations. Nevertheless, unforeseen scenarios may still pose challenges. Future work could explore more advanced augmentation strategies, such as physics-based simulations (Ma and Liu, 2020) or generative models, to further improve model generalization.

The current methods for sampling and feature fusion are based on experience and could be optimized. To be more specific, the number of splitters in data sampling (currently 20 per plot) and how we combine the 20 feature maps of sample images for each plot is worth further exploration and may provide an improved method for sample collection. The use of the 20 feature maps was done to avoid overcounting of seeds and was calculated based on the coverage of the camera and speed of the robot. This is an imperfect system due to the rugged terrain the robot operated in. While operating the robot, wet soil conditions may have caused slippage or accidental crashes into plots that would impact the average speed through any given plot and affect the accuracy of alignment of these 20 feature maps for certain plots. One benefit of using this system is that it allows each plot to be seen from every angle. In other words, we have four sets of feature maps. In our two-row plot configuration, each side of each row is imaged. This was done to ensure that every seed on the plot is viewed from at least one angle, as seeds only viewed from one angle may not be visible due to occlusion from other plant features such as stems or pods. While this reduces the chance for undercounting, it may lead to overcounting issues in cases where seeds are imaged from both angles.

To improve our approach, additional factors such as seed size and weight could be incorporated. Researchers have developed a transfer learning approach to automatically detect seed size in controlled imaging environments (Yang et al., 2021). Other researchers have expanded on this research by using imagery of soybean pods to detect pod width and length (Yang et al., 2022; Ning et al., 2022). Including these additional data points would provide a more comprehensive yield estimation model, improving the accuracy of selection decisions by capturing key phenotypic traits that influence overall yield. Utilizing additional data points, such as vegetative indices from hyperspectral imagery and soil conditions, could also provide additional improvements in a yield estimation model (Gupta and Singh, 2023; Chattopadhyay et al., 2023).

Unmanned aerial vehicles (UAVs) also play a vital role in modern yield prediction for breeding plots. Due to their rapid field-sensing capabilities, UAVs can survey numerous plots within minutes, making them an efficient tool for in-season data collection. Previous studies have demonstrated the effectiveness of using vegetative indices (Maimaitijiang et al., 2020), canopy texture data (Alabi et al., 2022), and canopy area (Yu et al., 2016) as inputs for machine learning-based soybean seed yield prediction models. Integrating in-season UAV scouting with our late-season seed detection and yield estimation model could potentially enhance yield prediction accuracy. However, UAVs often operate at heights over 30 m, which limits the visibility of lower plant regions and lacks the ability to sense fine details, such as individual seeds within pods, due to canopy overlap. Recent advancements, using UAV imagery angled at 53°–58°from approximately 4 m, have shown promise in detecting soybean pods (Li et al., 2024). This suggests that future improvements in camera technology could make UAV platforms compatible with high-resolution models like P2PNet-Yield, thereby decreasing the time needed to scout a field.

Operating at a speed of 4 kph, the TerraSentia robot has a battery life of approximately 2.5 to 3 h. With a 2-TB onboard storage capacity, the robot is capable of storing approximately 12 h of collected data before requiring offloading to an external storage device. Tasks such as recharging, battery swapping, and data offloading add time to the data collection process and limit the number of plots an operator can image per session. To streamline and integrate these processes, our proposed data collection pipeline could be embedded within a farm-wide data network, utilizing edge and cloud computing to automate data offloading and potentially enable real-time yield estimation (Singh et al., 2024). Citizen science networks have already shown the effectiveness of collaborative efforts in enhancing crop management and plant health (Chiranjeevi et al., 2023). Establishing farm networks can provide farmers with comprehensive information on optimal crop management practices. Deploying our model in a networked setting would allow it to be trained across more diverse genetic material, thus enhancing both the generalizability and accuracy of our model. By incorporating a wide range of genetic diversity and deploying our model across multiple environments, our yield estimation framework could significantly contribute to increased genetic gain (Krause et al., 2023).

Previous research has utilized depth cameras to detect soybean pods (Mathew et al., 2023). A promising direction for future work would be to implement this approach as part of a two-stage system: the depth camera could first locate pods, and then our P2PNet-Yield model could focus specifically on these areas to identify seeds within the pods more accurately. Similar 3D imaging techniques, such as LiDAR, have been used to create full 3D reconstructions of soybean plants (Young et al., 2024, 2023). Integrating our yield estimation model with these 3D plant structures could support breeders in developing enhanced soybean varieties, enabling the creation of an optimized crop ideotype for breeding (Singh et al., 2021d).

## 5 Conclusion

Traditional yield data collection methods are often costly, time-consuming, and susceptible to equipment malfunctions and maintenance issues. Our research introduces an innovative pipeline that streamlines the yield collection process, offering a more efficient and cost-effective solution for yield estimation and analysis. Specifically, we propose the P2PNet-Yield for seed yield estimation in soybean plots. We developed an end-to-end pipeline that demonstrates the effectiveness of ground robots for high-throughput soybean imaging, seed counting, and yield estimation. Additionally, we illustrated the application of seed counts and yield estimation for effective variety selection, particularly in discarding poor-performing varieties. In the future, as these ground-robot-based imaging platforms and DL methods continually improve, plant breeders will be able to forego combine harvesting at some locations and instead use P2PNet-Yield or related systems to obtain seed yield data.

## Data Availability

The raw data supporting the conclusions of this article will be made available by the authors, without undue reservation.
