# Supplementary material for: Robust soybean seed yield estimation using high-throughput ground robot videos
Source: Front Plant Sci. 2025 Mar 31;16:1554193. doi: 10.3389/fpls.2025.1554193 (PMC11994694; doi:10.3389/fpls.2025.1554193)
Supplement: Supplementary file 1 [file Presentation1.pdf]

Robust soybean seed yield estimation using high-throughput ground robot videos - Supplementary Material

1 SUPPLEMENTARY FIGURES

1.1 Improvement of Seed Counting Model's Performance on Corrected Fisheye Images

To illustrate the enhancement in seed counting performance with corrected fisheye images, we present seed detection results using two different sets of weights: the original weights and those obtained from training the model on our improved datasets. Figure 1a shows the results with the original weights, where the model struggles to detect seeds in the corrected fisheye images. In contrast, Figure 1b demonstrates that the model with the fine-tuned weights successfully detects most seeds in the foreground. This comparison highlights the significant improvement achieved through fine-tuning the model on the enhanced dataset.

1.2 Residual Plots of Models Trained on Different Combinations of the Datasets

The plots of residuals with ground truth seed counts show that the model trained on `MIX_AUG`, i.e., mixed datasets with data augmentation, performs the best. The residuals were distributed in a narrower band around 0 and did not show any deviation (Figure 2).

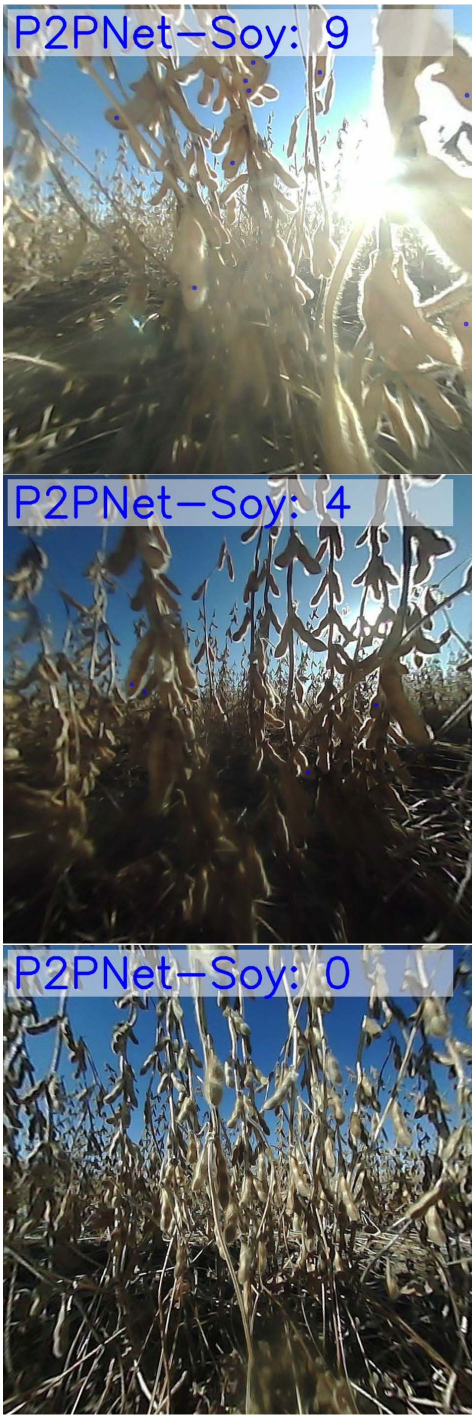

Figure 1a. Using original weights

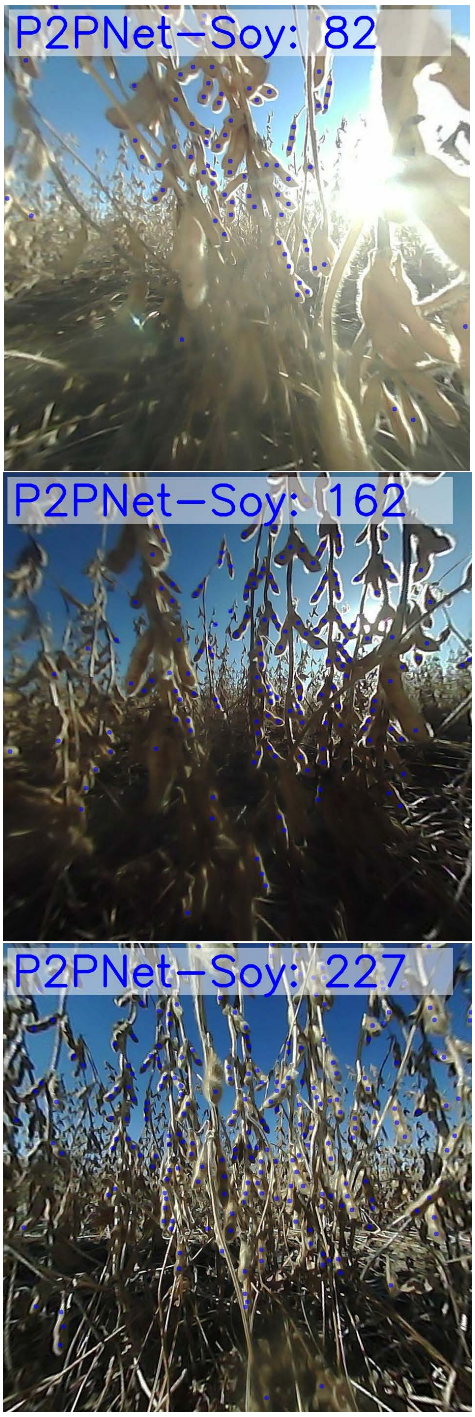

Figure 1b. Using our weights.

Figure 1. Comparison of seed detection results using original weights and our weights. **(a)** Using original weights; **(b)** Using our weights.

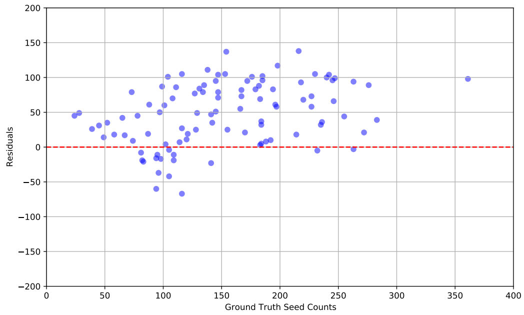

Figure 2a. ISU_NO_AUG

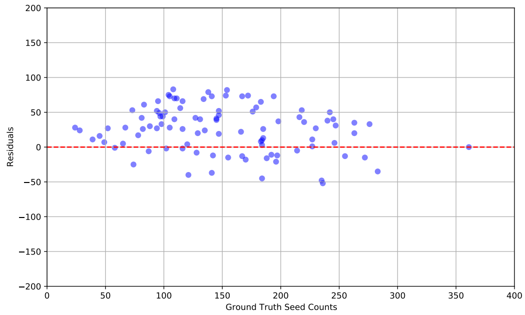

Figure 2b. MIX_NO_AUG

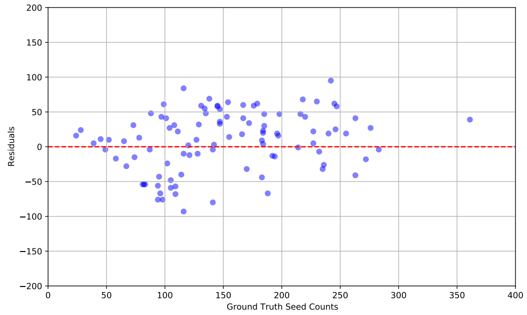

Figure 2c. ISU_AUG

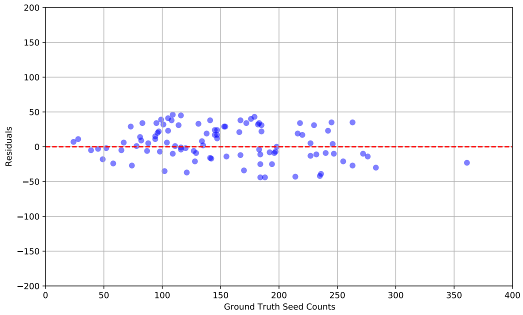

Figure 2d. MIX_AUG

Figure 2. Residual plots of models trained on different combinations of the datasets. The combination details can be found in Section 2.2. Results show that the model trained on mixed datasets with data augmentation performs the best.
